# The Genetic Data Market: Institutional Governance of Academic/Industry Research Partnerships for the Public Good

**DOI:** 10.1080/15265161.2025.2554770

**Published:** 2025-09-23

**Authors:** Kayte Spector-Bagdady

**Affiliations:** University of Michigan

**Keywords:** Genetics, industry, data, AI, public/private partnerships

## Abstract

The Trump Administration’s cuts to research funding and opposition to diversity, equity, and inclusion is destabilizing academic research. These attacks coincide with pointed government support for the private sector. But the conceptualization of academic versus private sector health research has historically been a false binary. Drawing on mixed methods research, this paper examines the genomic data market as an example of advantages and challenges of commercializing academic expertise. It also highlights the structural downsides of researchers individually navigating industry partnerships. While academia is currently being put in the unenviable position of being more likely to need the private sector to conduct research, with less federal funding to offer in exchange, structural pain points have existed for decades. This is an opportunity for academia to harness its powers of expertise and collective action to develop institutional policy to ensure academic/industry research is beneficial to the public health and diverse patient communities.

## INTRODUCTION

An opening salvo of the Trump Administration has been a seismic divestment of federal funding from the academic research enterprise (Holland 2025) and an aggressive attack on anything “diversity, equity, and inclusion” (DEI)-related (Department of Health and Human Services 2025; The White House 2025a, 2025b). To put too fine a point on it, Vice President Vance gave a 2021 speech called “The Universities Are the Enemy” (Goldberg 2025). Academic researchers and leaders will have to reassess approaches and priorities if they are to survive and thrive. One such approach, though not without tension (Marks 2019), is close collaboration with the private sector. For example, academic/industry research relationships in the genomic data market have been mutually beneficial for over a decade (Jaffe et al. 2024; Spector-Bagdady et al. 2024; Trinidad et al. 2023).

The current attacks on academia (Nadworny 2025) coincide with growing government support for the private sector (Vogel 2025). For example, in January 2025, President Trump facilitated a $500 billion private sector investment in infrastructure for artificial intelligence (AI). “Stargate”—a joint venture of OpenAI, SoftBank, and Oracle—plans to open data centers across the country and power the analyses of medical record data (Holland 2025). In July, the Trump administration announced a new partnership with companies including Google, Apple, and Amazon. Their goal is to facilitate people sharing electronic medical record (EMR) and other health information with private industry for reasons including AI development (Seitz 2025). Elon Musk, the former director of the U.S. Department of Government Efficiency (DOGE), owns his own private AI company (xAI) and also infamously disdains higher education (Aratani 2020). Meanwhile, the Trump administration has canceled thousands of federal research grants to universities with the current total at about $11 billion (Nadworny 2025).

But this proffered sharp line between academia and the private sector has historically been a false binary: that political leadership can take support away from academia *and* strengthen the private sector—that it is a zero-sum game between the two. Academic health researchers and industry players already work effectively together (Antman et al. 2017; Blume-Kohout 2012; Tierney, Meslin, and Kroenke 2016). For example, a recent study found that between 2010 and 19, NIH funding contributed to 99.4% of drugs approved by the U.S. Food and Drug Administration (FDA) for a total of $187 billion; an amount equivalent to that invested by the private sector itself (Galkina Cleary et al. 2023). Universities also intellectually contributed to patents underpinning 50% of FDA-approved drugs from 2020 to 24; 87% of those advances came from academic institutions in the United States (Gardner and Kinch 2025). Academia has leverage in these negotiations not only because it has historically been a conduit of federal research funding, but also because of the expertise carefully cultivated in its faculty, staff, and trainees.

Much past research on academic/industry collaborations has focused on conflicts of interest when academics receive funding or potential revenue from the drug and device industry. This research has found that studies sponsored by industry are significantly more likely to report benefits of a product, compared to those that are not sponsored (Lundh et al. 2018). And, while academics can theoretically offer unbiased assessments if their funding is not tied to a specific outcome (Tierney, Meslin, and Kroenke 2016), they can become just as biased as industry when they receive funding from it. For example, academic faculty with industry relationships are twice as likely to choose research topics with commercial advantages (Blumenthal et al. 1996). Many current academic/industry research relationships are different, however. They are based on health and health proxy data that can be accessed and shared in increasingly fast and novel ways, as well as access to the computing infrastructure necessary to enable machine learning and big-data analytics.

Without discounting the unprecedented nature of many of the current changes, academia does not have to approach this shift in a vacuum. While federal funding for academic research is currently seeing a steep decline, the expertise that academia possesses to accomplish such work remains a valuable asset. Academia and the private sector can continue looking to the other for collaborative advantage. And academia should collectively leverage that expertise via institutional policy to ensure research continues to achieve public health goals, such as health equity.

A full exploration of exactly what such policies should look like are outside the scope of this paper; they are debated extensively elsewhere (McCoy et al. 2023; Pike 2020; Spector-Bagdady et al. 2023). This paper will focus on the genomic data market as an example to explore advantages and challenges attendant to the commercialization of academic expertise. It will also assess the strengths and weaknesses of academics negotiating such relationships individually, rather than relying on collective institutional bargaining power. It is based on mixed methods research conducted from 2020 to 2022, which included 23 interviews with U.S. academic genetic researchers (Spector-Bagdady et al. 2024; Trinidad et al. 2023) and a survey of almost 300 more (Jaffe et al. 2024; Greene et al. 2025). These findings emphasize the symbiotic nature of academia and the private sector, but also the continued need for institutional policy to enable the collective academic action necessary for improving the health of diverse patient communities.

## THE GENOMIC DATA MARKET

The genomic data market consists of large, accessible, existing databases which can be critical for academic genetic researchers who do not have the time, money, and/or expertise to build such databases individually. Such databases are often held by government (e.g., UK Biobank, All of Us, dbGaP), consortia (e.g., 1000 Genomes Project, ENCODE), or private data stewards (e.g., 23andMe, Ambry, Ancestry DNA), or a combination thereof (Jaffe et al. 2024). These existing databases can centralize the time-consuming tasks of data collection, cleaning, and sharing. As one genetic researcher put it, “Collecting data…is one of the most horrific experiences known to man…other peoples’ old data [are] really much, much better…” (Trinidad et al. 2023).

Without access to these kinds of existing databases, academic researchers frequently lack statistical power to test their hypotheses. The size of a database is associated with frequency of use, especially for Genome-Wide Association Studies (GWAS) that rely on comparisons among thousands of datapoints. Establishing a relationship with a specific representative of a database also nurtures its long-term use. These relationships often remain strong as researchers train their students, postdocs, and junior colleagues in such collaborations—or even move to a different university. Using existing data can enable academics to focus on research, rather than tasks that they may regard as a wasteful use of their time and expertise (e.g., “annoying data cleaning tasks”) (Trinidad et al. 2023). Generating genomic data can also be expensive, something especially problematic for junior researchers with less external funding, compared to generally free access to existing databases (Jorgenson, Wolinetz, and Collins 2021; Trinidad et al. 2023).

Using federal funding from the National Institutes of Health (NIH) to generate genomic data *de novo* can also trigger data sharing requirements. While well intended and designed to promote the advancement of science, genetic researchers report that cleaning and organizing data to meet such requirements can take an inordinate amount of time (Greene et al. 2025). And, while the NIH allows grantees to spend funds to cover the “effort” required to data share, several problems remain.

For example, while funds can be earmarked to cover the cost of data sharing, the funds are not additive—if researchers need to save funds for data sharing, they cannot otherwise use it for data analysis or other work. In addition, grants often fund the years when data are generated, but end before final publications and data are posted. Funding during the project period for data sharing may be exhausted by the time most of the work needs to be done to clean and deposit data. Consistent with existing literature (Ali-Khan, Harris, and Gold 2017), we also found that genetic researchers are concerned about getting “scooped” or “beaten to the punch” if they are require to share data before they are finished publishing their own work (Spector-Bagdady et al. 2024).

The opportunity cost of time spent data sharing for NIH-funded work is also critical. Data cleaning and sharing requires a high level of technical aptitude and familiarity with the data. Senior faculty, who often prefer to spend their time on research, might assign these tasks to junior researchers or post-doctoral fellows. But time spent on data sharing often has little academic value to the individual actually doing it (Ross, Waldstreicher, and Krumholz 2023). Unless it is codified and recorded (and there are some mechanisms for this), it does not go on one’s CV, or factor in promotion. This means that sometimes those researchers who structurally most need to focus on career advancement are tasked with preparing data to be shared. As a genetic researcher observed: “Keeping our labs motivated, keeping our post docs motivated, keeping them productive is hard enough—and then having [to make] them go through some really cumbersome process to make their data available? Which involves both bureaucratic work and work organizing and curating the data, which people don’t often see benefit from?” This is likely related to the fact that many genetic researchers report that data from government databases, particularly those that have been shared under NIH data sharing mandates, often lack comprehensiveness. Researchers complain that others upload “just a fraction of the data” necessary for meaningful analysis (Spector-Bagdady et al. 2024).

These findings are consistent with work over the past several decades regarding limited data sharing between geneticists. For example, Eric Campbell and colleagues found that over 10% of geneticists reported intentionally withholding data from others for remarkably similar reasons over 20 years ago:

…80% reported that it required too much effort to produce the materials or information; 64%, that they were protecting the ability of a graduate student, postdoctoral fellow, or junior faculty member to publish; and 53%, that they were protecting their own ability to publish (Campbell et al. 2002).

The fact that data sharing has largely moved from an individual-to-individual system to a system built on centralized databases, seems not to have changed attendant academic disincentives.

Despite advantages, a critical problem with using existing data is a pervasive lack of data diversity. This can limit the generalizability of findings, or the ability to conduct research specifically tailored to improving the health of historically excluded communities (Bentley, Callier, and Rotimi 2020). In our interviews, some genetic researchers speculated that private databases, as opposed to government or consortia-controlled ones, are less likely to represent diverse ancestries because of self-selection biases (Trinidad et al. 2023). But our survey did not find any significant differences between ancestral representation across private, government, and consortium data stewards—all struggled with representation of non-European populations in numbers adequate to support genetic research (Jaffe et al. 2024).

Sometimes we cannot even measure database homogeneity. Robust demographic information is often not generated at the point of collection (Jiang et al. 2024). In one recent study of 28,000 original research articles being used to develop medical artificial intelligence (AI), authors found that under half reported sex, and less than 10% reported race and/or ethnicity. When race was reported, under 30% of participants were not White (Elmahdy and Sebro 2023).

We also know that most GWAS are conducted with participants of European ancestry (Popejoy and Fullerton 2016; Sirugo, Williams, and Tishkoff 2019), who represent 80% of global GWAS databases (Martin et al. 2019). Only 2.4% of participants in the National Human Genome Research Institute-European Bioinformatics Institute GWAS Catalog are of African ancestry, as compared to 78% who are European and 9% of East Asian descent (Morales et al. 2018). This can make contributors of European ancestry the “preferred cohort” for genomic research because they are so well characterized. They often serve as the benchmark for comparisons. Researchers with novel findings in populations of non-European ancestries might face even greater challenges in convincing others that their findings are valid, simply because their results are different than the Euro-centric baseline (Bentley, Callier, and Rotimi 2017).

This is consistent with our findings from interviews with U.S. academic genetic researchers. When we asked how they selected a genomic database for their work, not one brought up demographic diversity *de novo* (although, when pressed, almost all agreed it was important) (Trinidad et al. 2023). This qualitative finding was in contrast to our survey, which found the vast majority of genetic researchers report they aspire to work with data from non-European ancestries (Jaffe et al. 2024). The apparent conflict between findings is likely due to how the question was posed: if genetic researchers are directly asked whether they are *interested* in working with diverse ancestral populations, they overwhelmingly report in the affirmative. But, if they are open-endedly asked what they are actually *looking for* in selecting a database, none bother reporting data diversity—because they do not see it as a realistic option to begin with.

If genetic researchers must use large, homogeneous, existing databases for their research, the demographics of their participants can only reflect those of the overall pool (Trinidad et al. 2023). This lack of demographic diversity in databases is also reflected in the academic literature. U.S. academic genetic researchers are much more likely to publish data from participants of European ancestry as compared to any other background, including Indigenous, Arab or Middle Eastern, Asian, Hispanic, African, or mixed ancestries. The gap is particularly pronounced for data derived from Native Pacific, Pacific Islander, and other Indigenous populations (Jaffe et al. 2024).

When individual genetic researchers try to recruit ancestrally diverse participants, they can also face problems intersectional with those encountered generating data *de novo*. Long-term engagement with diverse communities can help build trusting, symbiotic, community-based research relationships (Benjamin 2014; Dang et al. 2014; Isler et al. 2013; Millon Underwood et al. 2013; Yu et al. 2013). But such community engagement also requires time and money, which can be limited on an academic grant and promotion timeline (Trinidad et al. 2023). And, for example, while the federal government’s *All of Us* research cohort is demographically diverse (Bianchi et al. 2024), it exists independently, outside of other, larger, databases. Generally, it also has a more comprehensive consent structure. While that is an intentional feature, given the increased focus of *All of Us* on community engagement and consent (which likely contributed to its success in *building* the database), these features can also act as a bug for researchers attempting to *use* it in harmonization with other data to amass significant cohorts (Jaffe et al. 2024).

“Generalizable” samples designed or weighted to proportional representation of the U.S. population alone will not solve this problem (Gao, Sharma, and Cui 2023). Simply increasing representation in databases does not necessarily equate to samples large enough to adequately support genomic analyses; weighting statistics cannot always result in generalizable data for minority subpopulations. And, even when the federal government *had* the goal of eradicating “health data poverty,” we were in a diverse health data deficit (Ibrahim et al. 2021). The current re-direction of federal funding away from working toward health equity (The White House 2025a; Wosen et al. 2025) will exacerbate these problems.

As Shawneequa Callier has argued, structural racism and the academic incentivization structure have encouraged genetic researchers to “collectively compromise equity in favor of values like efficiency, speed, and prediction accuracy” (Callier 2025). From the individual perspective, academic requirements for publication can not only impact promotion but also continued employment. Such requirements can incentivize the use of data from European ancestries because they are more efficiently accessed. In addition, higher-ranking journals are also more likely to prioritize the publication of large datasets (Callier 2025). In order to achieve such sample sizes, researchers trying to focus on non-European populations might need specialized tools and training to harmonize data across different databases (Trinidad et al. 2023). These needs might be increased by the additional complexity of studying participants with African ancestry, given their vast genomic diversity (Bentley, Callier, and Rotimi 2017).

While it has been normatively argued that investing in under-represented scientists will result in increased research with diverse ancestral populations (Oh et al. 2015), we did not find an association between researcher self-identified race or ethnicity, gender, or seniority and working with non-European populations (Jaffe et al. 2024). Of course, investing in underrepresented researchers in medicine and science has many benefits, explored elsewhere (Bentley, Callier, and Rotimi 2020; Guerrero, Huerta, and Pourat 2025). Yet our findings underscore Callier’s point that, from an individual researcher perspective, data *availability* is a major driver of use and advancing data equity is a collective action problem.

## HOW RESEARCHERS CHOOSE AMONG DATABASE STEWARDS

In addition to the genomic data market being able to offer some advantages for academic researchers generally, we also found that researchers often have a *choice* between databases—a choice which can have an important impact on their academic output. Overall, we found that data that are readily available, rather than those that respond to the most pressing public health needs, seem to heavily drive traffic. When asked what considerations they take into account when selecting a database, we found that researchers responded that they are more likely to prioritize upstream benefits of a collaboration (i.e. easy access or database features such as size) than downstream limitations on publication or data sharing (Greene et al. 2025). Such choices can also reflect academia’s systemization of prioritization of individual benefit over the public good.

### Private Database Stewards

U.S. academic researchers are increasingly using privately-held databases for their research (Spector-Bagdady et al. 2019). For example, while it has shifted types of corporate ownership (private, public, and now the not-for-profit TTAM), the direct-to-consumer (DTC) genetic testing company 23andMe possesses the largest genomic and phenotypic database for therapeutic research in the world (Prince and Spector-Bagdady 2025). It has over 15 million consumers, the vast majority of whom consent to research. Consumers have also contributed over 4 billion self-reported “health and trait data points, including disease diagnosis, medication use, medication side effects, lifestyle, diet, and family health history” (23andMe 2024).

23andMe, along with Color Genomics and Gene by Gene, dominate the global $928 million DTC genetic testing market (Research and Markets 2018). The databases of private genotyping services are generally built through three main models: (1) consumers purchasing DTC testing directly to receive results regarding their ancestry or health, (2) clinicians ordering genetic testing for their patients and sending the tests to industry for analysis; and (3) academic researchers recruiting participants through an academic/industry genetic testing partnership. As an academic researcher argued, DTC genetic testing companies “leapfrog over everybody” because they get *paid* to collect data and “they don’t have to get all their grants rejected all the time and keep reapplying. They’re not begging for money, the way people in academia are, to do this kind of research” (Trinidad et al. 2023).

23andMe reports data sharing agreements with over 150 pharmaceutical and biotech companies as well as major academic medical centers (AMCs) such as University of Chicago, the Broad Institute at MIT and Harvard, and Stanford University (23andMe 2025). It prominently advertises these collaborations on its website so that it, as one academic researcher put it, “at least it looks like they are working with name brand institutions when other researchers are looking at the website to see whether they should work with them” (Spector-Bagdady et al. 2024), a finding echoing similar work by the National Research Council (National Research Council 2003). 23andMe also had a “Genotyping Services for Researchers” program where academic researchers can send in samples from their recruited participants for analysis. In this model, 23andMe provided researchers external genotyping, often less expensively than it would be to do it in-house, and participants received the incentive of return of ancestry results (23andMe 2016).

There is a reason that the private sector heavily markets the use of their databases to academic researchers: the collaboration with academic expertise can be a huge market advantage (National Research Council 2003). Private sector researchers can benefit from co-authorship opportunities, learning new research methods from academic colleagues, analyzing more data, and attracting new customers (Spector-Bagdady et al. 2024). AMCs can also offer patients for industry recruitment. Often large clinical trials must be conducted across several sites to ensure enough participants across a heterogeneous pool, which has been historically challenging for private industry to achieve itself (Bodenheimer 2000).

Being able to publish in prestigious journals with academic colleagues is also an advantage to the private sector (National Research Council 2003). As Thomas Bodenheimer has argued:

For academic investigators, publication in peer reviewed journals is the coin of the realm. For pharmaceutical firms, in contrast, the essential product is the new-drug application to the FDA. In the absence of FDA approval, no journal article is worth a cent to a drug company. Yet publication in prestigious journals is important, to persuade physicians to prescribe the company’s products (Bodenheimer 2000).

In genomic data partnerships with DTC companies, this advantage might even be more pronounced.

As mentioned, 23andMe’s phenotypic data come from consumer self-report via surveys in emails and on the 23andMe website. This is a far cry from the “gold standard” of clinical information captured by a healthcare professional in the EMR. Pharmaceutical companies researching and developing new drugs must meet strict FDA requirements for data accuracy, completeness, and reliability to submit their research in support of a new drug application. Originally, regulators voiced skepticism regarding the validity of self-reported data in supporting new product approvals (Offord 2017; Wyatt et al. 2013).

The *de facto* validation of self-reported data by publishing it in top-tier academic journals can therefore be a major market advantage to the private sector. As an academic researcher put it: “…it legitimizes 23andMe as a company, makes them look better in their research…they prove that the way they collect data is valuable, mainly by…self-report, and maybe that helps them build a case for then selling the data to various drug development companies” (Spector-Bagdady et al. 2024). For example, as the owners of the most samples from participants with Parkinson’s disease, in 2015 23andMe struck a $60 million deal with the biotech company Genentech for drug development (Herper 2015). DTC genetic testing companies are also seen as altruistic and “happy to see that their data is used for interesting scientific questions” (Spector-Bagdady et al. 2024). Academic collaboration can also allow companies to benefit from the “health halo” that can come from working alongside trusted research institutions (Marks 2019; National Research Council 2003).

Much like the use of federal funding toward new drug approvals discussed above (Galkina Cleary et al. 2023; Gardner and Kinch 2025), academic genetic researchers also often invest their NIH funding into private research collaborations (Greene et al. 2025). For example, almost half of academic researchers using privately-held genomic data cited NIH funding in their publications (Spector-Bagdady et al. 2019). Those NIH funds are used for things such as data analysis, personnel effort, data cleaning, data management, data collection, and data access (Greene et al. 2025).

But private data stewards are more likely than government or consortia stewards to limit data sharing at and after publication, even if academic researchers use their NIH funding in the collaboration (Greene et al. 2025). The private sector can also prevent publication in journals with data sharing mandates. If NIH funding is used to generate these data, such agreements can interfere with NIH data sharing requirements (Spector-Bagdady 2021). This behavior has been demonstrated in research outside of the genomic context as well (Blumenthal et al. 1997; Campbell et al. 2000). As Rebecca Eisenberg and Michael Heller have argued, this kind of upstream limitation in data sharing can impact the ability to conduct downstream research building on or replicating such data and can lead to underutilization of a valuable resource. They call this the “tragedy of the anti-commons” wherein “multiple owners each have a right to exclude others from a scarce resource and no one has an effective privilege of use” (Heller and Eisenberg 1998).

While the rapid rate of change in the academic/industry health data market may be surprising, its direction is not (Institute of Medicine 1994; Evans 2011; Wilbanks and Topol 2016; Spector-Bagdady 2016). If the federal government is going to invest its funding, and academia is going to invest its expertise, in building and validating large private databases, both must ensure that they additionally leverage these assets to the benefit of the public’s health.

### Government Database Stewards

While U.S. academic genetic researchers are increasingly using privately-held data for their research, they are still more likely to work with government data stewards in comparison (21% vs. 84%) (Greene et al. 2025). Working with government stewards offers different kinds of benefits. For example, government data stewards, as compared to private or consortia stewards, are the least likely to place restrictions on subsequent data use, publication, and sharing—like prior review or approval before article submission. Government data stewards are also the least likely to require co-authorship on resultant publications, a requirement that otherwise gives the data provider control over selection and interpretation of data (Greene et al. 2025). This is similar to past research finding that academic faculty who accept industry sponsorship are also more likely to report delays in publication (Blumenthal et al. 1997).

However, government databases sometimes require the researcher to have their own equipment and software to store data. This can be expensive and require new equipment or specialized training in how to use a centralized platform. Therefore, while data access is often “free” from government databases, access costs are sometimes not reflective of the cost of full data usage. As one U.K. Biobank researcher complained, “we needed to have the IT support to help us download all the data and arrange it and show us how to access it,” otherwise “the answers turned out rubbish.” Support for data management—including validation tools, harmonization, and storage—is therefore an enticing attribute of a centralized database. Relying on inexpensive or otherwise easy access to data without data management capabilities can actually “cost” an individual researcher more in the long run due to the price of downstream analysis (Trinidad et al. 2023). Selection of a data steward can therefore have a significant impact on academic research in genomics, affecting the data available, the type of research enabled, the publications supported, and the ability to share data (Greene et al. 2025).

## CONTINUED EVOLUTION OF THE HEALTH DATA MARKET

The health data market continues to rapidly evolve in ways that will impact academic/industry research partnerships. It has historically been assumed that AMCs (as opposed to the private sector) have the advantage of generating and holding gold-standard EMR data. In hospitals, not only do patients have a compelling interest in sharing their medical information, but clinicians are often reimbursed from health insurance to generate and capture it in detail. In 2017, for example, researchers found that AI machine learning tools were more likely to be trained on data from AMCs and other hospitals with a high level of data collection and processing resources (Tikkanen et al. 2017). And in 2020, researchers found that over 70% of AI algorithms were trained on health data sets from California, Massachusetts, and New York alone (Kaushal, Altman, and Langlotz 2020), due mainly to their high density of AMCs.^1^

But industry is increasingly collecting data from consumers and patients directly. Some companies have enough to even offer “shadow health records” for research. These records are built from health and health proxy data available on the internet about individuals and through data-brokers. IQVIA, formerly the data broker IMS Health, reports having “approximately 400 million comprehensive, longitudinal, anonymous patient records” and “85% of the world’s prescriptions by sales revenue” (Price et al. 2019).

There are also currently more than 337,000 digital health apps collecting data directly from consumers and over 100 digital diagnostics commercially available, many of which are AI enabled. Patient-facing digital tools include health and wellness apps, self-care support, and digital therapeutics. For clinicians, there are decision support tools as well as clinical and research platforms. Medication management and remote patient monitoring apps facilitate clinician/patient interactions (IQVIA Institute for Human Data Science 2024). With some of these tools, patients report their health information directly to a private company, without the involvement of a clinician. Others operate under the genetic data market model: clinicians order tests or monitor their patients using private sector platforms which then retain the data for their own research and commercial purposes. The private sector is also increasingly getting involved at the point of care itself. For example, the private company UnitedHealth acquired ownership in more than 100 outpatient surgical centers just last year (Herman 2025).

As the cost of genomic sequencing decreases, the generation of large, heterogeneous genomic databases is increasing. But being able to process, analyze, and interpret these data to their full potential has been historically challenging (Strudwick et al. 2024). The use of machine learning and AI for genomic and other health research is therefore also having a large impact on the market, shifting focus from just data *access* to its increasingly necessary partner: analytic computing power.

Machine learning technologies can now accelerate and advance genomic data analytics, predictive modeling, and patient care (Gao, Sharma, and Cui 2023). They can more efficiently identify genetic mutations linked to, and patients at high risk of developing, hereditary diseases (Mieth et al. 2021). AutoML-GWAS can compute individualized prediction scores (Lakiotaki et al. 2023). Vision-language models have made it possible to learn from medical imaging, clinical notes, and structured clinical data (Alayrac et al. 2022; Kline et al. 2022). These data can provide context when interpreting genomic risk markers and predicting outcomes (Luo et al. 2020). AI-genomics applications can also recommend clinical interventions and enable drug discovery (Gao, Sharma, and Cui 2023). Models that support physicians in decision making and diagnostics have already been integrated with EMR systems and provide ranked lists of potential diagnoses (Rehan 2023). As 23andMe advertised, “Bigger is better because as the size of 23andMe’s database grows and as we apply new technologies like AI and machine learning to this massive dataset, researchers can find more genetic associations, leading to more potential drug targets” (23andMe 2024).

But these machine learning-enabled genomic analyses require massive computing infrastructure—the kind that the private sector is overwhelmingly more likely to possess. For example, Graphics Processing Units (GPUs) are necessary to support deep learning and other advanced analytics, not just for images but for a wide variety of data-intensive tasks. GPUs are designed to perform thousands of operations in parallel, enabling large-scale, repetitive calculations required by machine learning algorithms applied to massive genomic datasets. The University of Michigan (U-M), one of the top NIH-funded R1 research institutions in the world, reports access to 300 GPUs (Information and Technology Services 2025). OpenAI has 16,000 GPUs, just in its Texas facility (M. Lee 2025). U-M recently announced a partnership with OpenAI as part of NextGenAI, a commitment by OpenAI to support academic research partnerships. It includes a $50 M research investment and academic access to OpenAI computing capabilities (Jordan 2025). As the OpenAI Chief Operating Officer announced: “The field of AI wouldn’t be where it is today without decades of work in the academic community. Continued collaboration is essential to build AI that benefits everyone” (OpenAI 2025). Particularly as data-driven research moves toward machine learning-powered analytics, access to computing infrastructure will increasingly determine what research gets done—and by whom.

## LIMITS OF CURRENT GOVERNANCE

Academic/industry health research partnerships have been occurring for decades, largely for the benefit of both kinds of institutions. The challenge is to ensure that choices made for mutual benefit *also* benefit public health—and the diverse patient communities that make it up (Marks 2019). Patient engagement and trust building are necessary to alleviate the public health burden of homogeneous data. Individuals make up the pool of data from which researchers draw, as well as the patient populations researchers hope will benefit from using such findings in the clinic. And patients are resoundingly skeptical of their data being “commercialized” or shared with the private sector.

The majority of patients agree patients should share health information and specimens for low-risk research (Jagsi et al. 2023). Patients are most willing to share with the hospital they attend, but willingness drops precipitously if the sharing is with outside commercial interests (Kim et al. 2019; Spector-Bagdady et al. 2018, 2022). Willingness to share data with academic institutions can increase in times of public health emergencies, like the COVID-19 pandemic, but remains low with commercial entities (Tosoni et al. 2022).

Patients express concerns about *genomic* data sharing specifically and are, again, consistently more concerned about sharing them with for-profit entities (McGuire et al. 2011; Middleton et al. 2020; Rosas et al. 2020). Willingness to donate for genetic research is also associated with several demographic variables including older age, socioeconomic status (lower for participants who identify as non-Hispanic White and higher for participants who identify as Black or African American), and identifying as non-Hispanic White as opposed to Black or African American, Asian, or Hispanic White (Middleton et al. 2019; Spector-Bagdady et al. 2021). These factors are not only associated with consent rates, but can also be associated with being less likely to be *recruited* to join a genomic biobank in the first place (Spector-Bagdady et al. 2021). Patients also express concerns regarding who profits from their genomic data or specimens, industry exploitation, and heightened privacy risks (S. S.-J. Lee et al. 2019; Spector-Bagdady et al. 2020). Many patients also have privacy concerns about AI use of their health data (Ellis et al. 2025; Robertson et al. 2023).

Current governance models are not alleviating such concerns. When academia and industry collaborate in the clinical trial space, FDA generally offers a layer of external oversight through its human subjects research regulations and product review (Bak et al. 2025). Such regulations are so strict that they can, ironically, even make academic federally-funded research more burdensome than private sector research (Spector-Bagdady 2021). But oversight of academia’s involvement with “big tech” is far less centralized due, in part, to limitations of FDA’s governance remit in devices generally and AI specifically (Price, Sachs, and Eisenberg 2022). FDA has authorized ~1,000 AI-enabled medical devices to date (the majority of which are in radiology). It has also received hundreds of new drug applications that rely on research using AI technologies. FDA leadership has expressed concern about its purview and ability to sufficiently review this many new uses and applications of AI as “the scale of effort needed could be beyond any current regulatory scheme…” (Warraich, Tazbaz, and Califf 2025). And that was before Trump Administration threats, firings, and cuts destabilized the agency; FDA recently lost almost 20% of its workforce (Lawrence 2025).

While there have been impassioned arguments for, and successful examples related to, self-governance in science (Maurer 2017), this approach has been roundly ineffective in the commercial health data sharing space (Pike 2020). As Jonathan Marks argued in his book, *The Perils of Partnership*, although the private sector “may well contribute to the public good,” they are not “guardians of this common good” (Marks 2019). And, as Wendy Parmet had to remind us during the COVID-19 pandemic, “Unquestionably, the private sector has a role to play in public health… But to rely on it to *protect* the public’s health is pure folly” (Parmet 2021, emphasis added). And, as Leah Fowler, Anya Prince, and Michael Ulrich recently pointed out: “Companies have collected and monetized personal data for years… There comes the point where the evidence against the technology industry’s willingness and ability to self-regulate becomes too overwhelming to ignore” (Fowler, Prince, and Ulrich 2023). Self-regulation does not appear promising.

## REFLECTIONS ON FUTURE GOVERNANCE AND FUNDING

While Fowler and colleagues concluded their 2023 paper with a call for improved health privacy legislation, given the fact that the Trump Administration’s AI Executive Order, “Preventing Woke AI in the Federal Government,” prohibits U.S. department and agencies from assessing AI models for “pervasive and destructive” biases such as sexism and racism (The White House 2025c)—that seems unlikely to be effective now. Luckily, statutes and regulations are but two tools in the health policy toolbox.

These relationships can also be influenced via the NIH’s “power of the purse,” including its ability to require federally funded actors to follow NIH policy. The NIH has used this power in the past to nudge and sludge public-private collaborations in directions that emphasize equity and public benefit (Britt 2021; Clark and Callis 2022). But this power can also fall short, as our findings regarding NIH data sharing policies conflicting with industry data use agreements have indicated (Spector-Bagdady et al. 2024). They will also almost certainly fall short in the near future, given the strict curtailing of NIH extramural funding (Thorp 2025).

I have, in the past, argued that the over $12B in NIH funding that AMCs receive gave them greater negotiation power than they were leveraging with the private sector in developing health data partnerships (Spector-Bagdady 2021). But now, the ability of academic researchers to negotiate in this way will likely diminish due to reduced federal funding for such collaborations (Thorp 2025), less privileged access to patient EMR data (Herman 2025), and decreased federal oversight (Lawrence 2025). As NIH funding and guardrails weaken, or disappear altogether, academia might be put in the unenviable position of being more likely to need the private sector to conduct its research (from a data, funding, and computing perspective) with less of its traditional negotiation power.

But academia’s most valuable asset is the expertise of academics themselves. The genetic data market is a clear example of its power. Even if academia cannot wield the incentives of money or patient data in the same way right now, they still control the incentive of academic expertise. And industry’s access to expertise can be limited by academic institutional policy.

Historically, there are already success stories of academic institutional policy impacting academic/industry relationships to improve the public health. For example, in the early 2000s, during increasing scrutiny of conflicts of interest caused by big pharma incentives to clinicians such as food and travel, Yale University, Stanford University, the University of California, Davis, and the University of Pennsylvania banned pharma sales representatives from campus in short order. Many other hospitals followed (Brennan et al. 2006; Kirn 2007; Malone and Richter 2006; Peart 2006). These bans lowered AMC clinician prescribing rates of drugs that had been previously “detailed” by sales representatives, and increased prescribing rates of drugs that were not—which were often less expensive generic equivalents (Larkin et al. 2017).

This is not to say that, if left to their own devices, individual academics will *not* make choices in line with patient values and public health. Many do every day. But *individuals* in the academic/industry data sharing space are not always enabled to make the kinds of choices necessary to have an impact at the population level. For example, and as discussed previously, scholars have suggested that increasing the number of genetic researchers with historically excluded backgrounds will increase research with historically excluded populations. This makes sense if individual researcher preference and/or bias drives data selection; if you fund researchers with different preferences you will get a different result. There is certainly compelling evidence for such a hypothesis; individual and systemic discrimination—and in particular anti-Black racism—is pervasive in (and foundational to) American academic medicine (Fletcher et al. 2022; Nong et al. 2020; Pleasant et al. 2024).

The impact of individual preference on data diversity, however, was not what we captured in our work with U.S. academic genetic researchers. We found a very high rate of overall reported interest in research with historically excluded ancestries. We did not find an association between self-identified researcher demographic characteristics (i.e. race or ethnicity, gender, and seniority) and the diversity of ancestral populations represented in their work (Jaffe et al. 2024). While our own research certainly had limitations, and more work in this space is warranted, our findings are consistent with the context of the *structure* of academic medicine largely driving choice of database, rather than individual researcher preference or bias (Spector-Bagdady et al. 2024; Trinidad et al. 2023).

No matter the self-identified race or ethnicity of the researcher, if diverse data are not readily available, or do not harmonize with standard analysis tools, or are less likely to be published by high-impact journals, or need to be collected *de novo* over many years, barriers to achieving academic structural expectations of high-impact publications within a limited time frame remain consistent. As Callier has argued: “Bolstering the inclusion of diverse populations in health research datasets promises to mitigate underrepresentation but may be insufficient without a framework for evaluating the ethics of tradeoffs made throughout the course of research that cause investigators to disregard available data from underrepresented populations” in the first place (Callier 2025).

In fact, this likely makes it *harder* for researchers from historically excluded communities to work with data from patients from historically excluded communities because they themselves are facing intersectional barriers related to individual and systemic racism in their own careers (McFarling 2022; Nunez-Smith et al. 2007). From this perspective, expecting historically excluded researchers to increase research with historically excluded communities is but another example of minority or “cultural taxation”—placing the burden of resolving racism onto the very community impacted by it (Padilla 1994). It is academic structure that needs to change, and its collective bargaining power that needs to change it.

The fact that federal executive branch priorities are currently destabilizing the federally funded research enterprise is at least an opportunity to reconsider policies related to academic/industry partnerships. While it might be tempting to send individual academic researchers out to fund their work and salaries in whatever way possible, it would be short-sighted for academia to act as if it no longer has any leverage in industry negotiations, or that academics will be able to protect embattled public health goals individually. Academia should now exert its own power of expertise and collective action by requiring industry collaborations to comply with structural institutional policies that enable research generalizable to or directed for historically excluded communities.

## CONCLUSION

Academia is confronting an existential threat to its long-term sustainability. Lessons from the genetic data market can help inform effective steps forward. While research funding might be in steep decline, academia still retains the expertise that has made it a valuable industry partner for decades. While academics can broker assets individually, doing so in isolation makes it challenging to achieve critical public health objectives, like equity. History has demonstrated, however, that collective academic action can drive meaningful public health improvement. By bargaining its aggregate expertise in exchange for compliance with thoughtful institutional policy, academia can not only bolster its research financial stability but also advance equitable public benefits from academic/industry research partnerships.

## Footnotes

1 The author would like to thank Dr. Amit Kaushal for providing the underlying data from his 2020 article to support this additional sub-analysis.
